# Spatial and temporal changes of outdoor thermal stress: influence of urban land cover types

**DOI:** 10.1038/s41598-021-04669-8

**Published:** 2022-01-13

**Authors:** Mohammad A. Rahman, Eleonora Franceschi, Nayanesh Pattnaik, Astrid Moser-Reischl, Christian Hartmann, Heiko Paeth, Hans Pretzsch, Thomas Rötzer, Stephan Pauleit

**Affiliations:** 1 Strategic Landscape Planning and Management, School of Life Sciences, Weihenstephan, Technische Universität München, Emil-Ramann-Str. 6, 85354 Freising, Germany; 2 Forest Growth and Yield Science, School of Life Sciences, Weihenstephan, Technische Universität München, Hans-Carl-von-Carlowitz-Platz 2, 85354 Freising, Germany; 3 Institute of Geography and Geology, Universität Würzburg, Am Hubland, 97074 Würzburg, Germany

**Keywords:** Environmental impact, Plant ecology

## Abstract

Green infrastructure (GI) has emerged as a feasible strategy for promoting adaptive capacities of cities to climate change by alleviating urban heat island (UHI) and thus heat stress for humans. However, GI can also intensify the winter cold stress. To understand the extent of UHI within a city as well as the link between outdoor thermal stress both diurnally and seasonally, we carried out an empirical study in Würzburg, Germany from 2018 to 2020. At sub-urban sites, relative humidity and wind speed (WS) was considerably higher and air temperature (AT) lower compared to the inner city sites. Mean AT of inner city sites were higher by 1.3 °C during summer and 5 °C during winter compared to sub-urban sites. The magnitude followed the spatial land use patterns, in particular the amount of buildings. Consequently, out of 97 hot days (AT > 30 °C) in 3 years, 9 days above the extreme threshold of wet bulb globe temperature of 35 °C were recorded at a centre location compared to none at a sub-urban site. Extreme heat stress could be halved with 30–40% cover of greenspaces including grass lawns, green roofs, and green walls with little compromise in increasing winter cold stress.

## Introduction

Networks of green and blue open spaces, i.e. urban green infrastructure (UGI), have emerged as feasible strategies to promote adaptive capacities of cities to climate change, particularly to more frequent and intense heat waves and flash floods from rainstorms^1–3^. The term green infrastructure (GI) has been defined variously to slightly different concepts. However, this term is generally used for denoting a strategically planned and managed network of natural and open spaces to deliver a wide range of benefits or ecosystem services (ES). UGI can provide a wide array of ES to the residents i.e., regulating (such as thermal regulations), provisioning (such as food) and cultural services (such as recreation). As an adaptive strategy, trees are mostly emphasized at local level to reduce thermal stress^4–6^. However, at landscape level urban morphology and geometry or the size and the shape of urban spaces, for instance, the building height to street width ratio (street canyon aspect ratio H/W) are equally important^7^ along with the presence of urban trees. In heterogeneous urban ecosystems^8^, trees have multiple biophysical functions, among them radiative shading and evapotranspiration are predominant^9^. Firstly, with their extended canopies, trees reduce the input of shortwave radiation to the ground level by up to 90% ^10^, in particular during summer when deciduous trees are in leaf in temperate and cold climates^11^. Secondly, through transpiration trees cool their immediate vicinity between 1 and 8 °C^12,13^ and consequently increase the relative humidity of the air^13^. Nevertheless, depending on the urban space design, trees can have reduced evapotranspiration under soil drought and higher temperatures conditions^11^. At the same time, trees can also bring some negative effects such as hindering the vertical turbulent mixing within narrow street canyons and breeze—horizontal advection^14^. Whereas, grass cover mostly reduces the radiative heat load by higher reflection compared to built environment and evapotranspiration^15^ but allows higher wind speed to reduce summer heat load and greater solar radiation for reducing winter cold stress.

The urban heat island (UHI) effect, i.e. the warming of cities compared to their surroundings^16^, in particular during the summer season, appears to be one of the major climate challenges in Central European cities^17^. While UHI intensity can be stronger in winter compared to other seasons^18^, summer heat island poses a negative impact on the human use of public spaces and overall on the health of the population. Oudin Åström et al. ^19^ analysed the excess daily mortality of the 50+ population in the Mediterranean City of Rome and reported a 22% increase during heat waves as compared to normal summer days. Therefore, cities have invested considerable resources to mitigate the adverse impacts of the summer heat island^20^ such as the covenant of mayor initiative (https://climate-adapt.eea.europa.eu/eu-adaptation-policy/covenant-of-mayors) of some big cities such as Portland, Copenhagen, New York that are leading at the global scale through planting trees, installing green roofs, reflective pavements etc. Yet, most of the current adaptation strategies focus on reducing the daily maximum air temperature while ignoring the nighttime thermodynamics of cities^21^. On the other hand, the surface energy balance of the built environments can provide significant benefits to urban dwellers by reducing the heating demand during the winter ^20,22^. Therefore, it is important to consider the seasonal and diurnal variability of air temperature when applying the knowledge of outdoor thermal comfort as a planning tool to consider tradeoff and further base the planning knowledge.

Reduction of heat risks and enhance outdoor thermal comfort while promoting physical activity and wellbeing in urban areas are very important, since the majority of the population of today’s world live in cites ^23^. For quantification of thermal stress over 60 heat stress indices have been proposed, with their own advantages and limitations^24^. Among the indices, physiological Equivalent Temperature (PET) is one of the most commonly used indices for measuring heat stress in outdoor spaces, which is based on the output of Munich Energy Balance Model for Individuals (MEMI)^25^. Another commonly used index, universal thermal comfort index (UTCI) originates from an approach that was proposed over 10 years ago by the International Society of Biometeorology (ISB) Commission. On the other hand, Wet Bulb Globe Temperature (WBGT) suggested by Yaglou and Minard ^26^ (ISO), has the advantage of being based solely on actual environmental variables. Four parameters, dry temperature, relative humidity, wind speed, and radiation heat are considered in more detailed manner and present a more accurate value in comparison with other heat indices^27^. In a recent study, Zare et al. ^23^ attempted to correlate different thermal indices using 1 year of meteorological data and reported correlation coefficient of 0.96 between UTCI and PET and 0.88 between UTCI and WBGT. Many occupational and sport safety recommendations are based on the WBGT indices. For instance, a review of health guidelines by Epstein and Moran ^28^ noted that for a WBGT range of 25.6–27.7 °C, sustained outdoor work of at least 4 h can be maintained using a cycle of 40 min work and 20 min of rest. However, with an increase of WBGT by ~ 6 °C (> 32.2 °C), there is a drastic change in the cycles, namely − 10 min work and 50 min rest. This implies that a substantial shift in the working schedule is required under future climate scenarios if no countermeasures were taken.

Improving thermal comfort is one of many adaptive planning targets to mainstream resilience, but an important one as climate is warming and greenspaces are shrinking due to increased impervious cover and drought stress^29,30^. For analyzing the sensitivity of parameters for determining outdoor human thermal comfort, data on urban built surfaces can be more easily modeled, but parameters related to greenspaces are hard to simulate. Along with the increase of wind speed^31^ the cooling benefit of street tree canopies usually increases within broader and shallow street canyon conditions. In any case, the interaction of built environment and greenspaces are complex. Lehnert et al. ^32^ assessed the universal thermal climate index (UTCI) on selected summer hot days of 2018 and 2019 across nine sampling points in four cities in the Czech Republic. They reported up to 10.5 °C reduction at sites with urban parks which consist of tall trees, shrubs and grass compared to sites with low vegetation, i.e. mostly grass lawns with a reduction of up to 2.3 °C UTCI. Similarly, during the dormant winter period, built environment rather can provide better outdoor thermal comfort to the city dwellers^20^. However, studies on the seasonal variations on thermal benefits as well as penalties of different land cover types such as convective heat from built surfaces during summer and blocking radiation by trees during the winter have not been investigated either using remote sensing techniques^33^ or using empirical ground-based techniques^5,20,33^. The cooling intensity of urban greenspaces are associated with the spatial heterogeneity of landscape, and is positively related to the composition, configuration and the size of the greenspaces^34^. Moreover, the efficiency of the cooling effect also might increase depending on the surrounding natural and semi-natural landscapes^35^. The present study addressed these knowledge gaps by exploring the relationships between different land cover types within a 500 m buffer zone around immobile meteorological stations with human outdoor thermal stress across different seasons at different spatial scales. This will also help to upscale the ground-based observation to link to landscape level. Two popularly used landscape metrics are landscape composition which indicates mainly the amount and type of land use, and landscape configuration which considers the geographic features of every land use. Both are very important since they affect the radiative and energy fluxes ^33^. Characterizing urban green space landscape patterns using these two landscape metrics in terms of mitigating the human thermal discomfort over the diurnal cycle and over the seasonal cycle, we need to better understand the tradeoff between low green cover versus treed space^36^.

To close the knowledge gaps, we carried out an empirical study in Würzburg, Germany. Würzburg is one of the major cities in Northern Bavaria, and one of the driest and warmest cities in Southern Germany with a substantial UHI effect with a daily intensity of up to 6 °C ^5,37^. Thus, the monitored data allowed to answer the following research questions.

1. What is the extent of urban heat island (UHI) within the inner city compared to the sub-urban areas both in a hot summer and a cold winter?
2. What are the variables, factors and co-variables for variations in the Wet Bulb Globe Temperature (WBGT) across spatial and temporal scales?
3. How to assess the interaction between UHI mitigation measures and cold-wave hazards in terms of land cover types?

## Methods

### Study area

**Figure 1:**
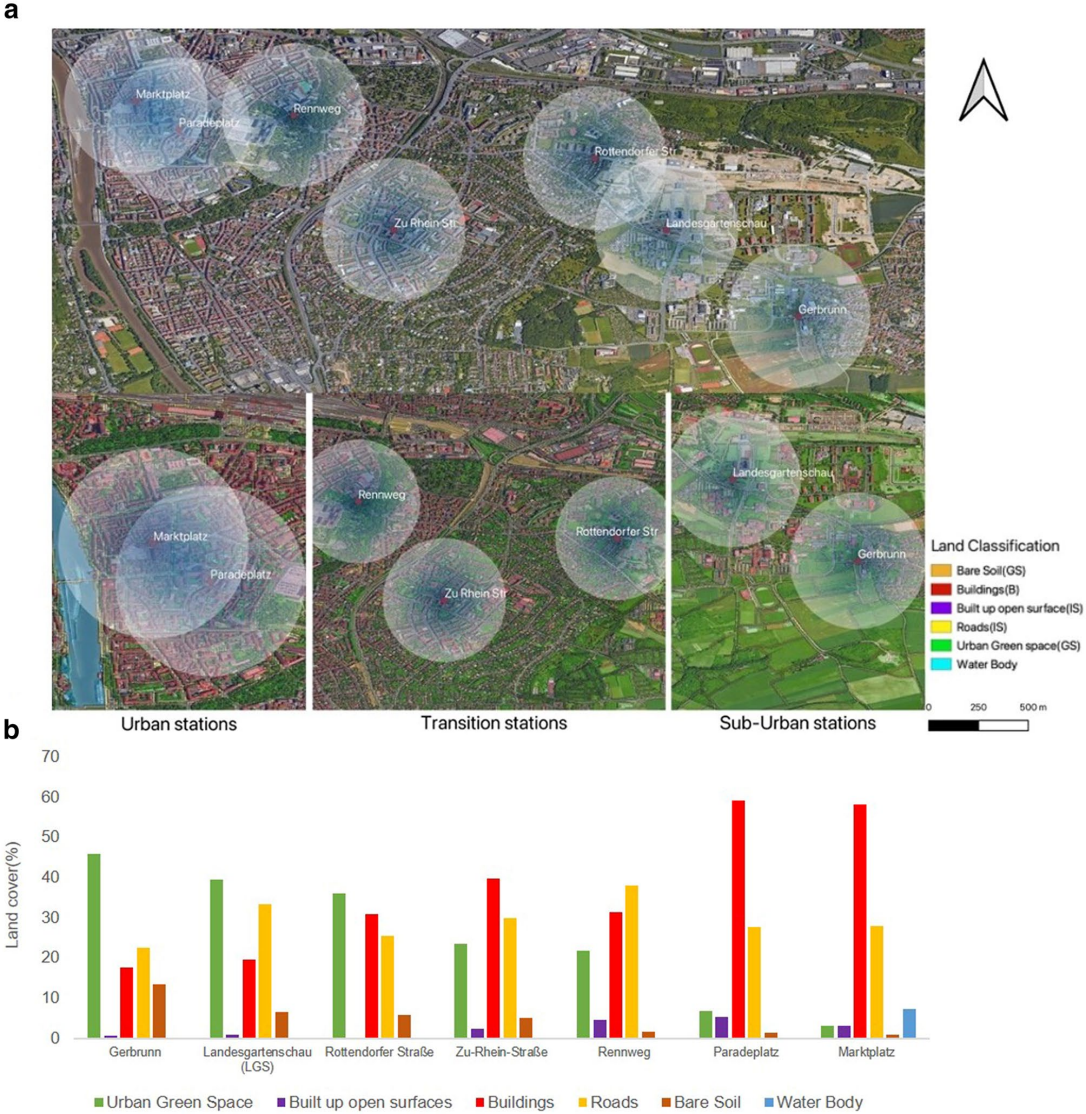
Study area—the city of Würzburg across the urban-sub-urban transect (**a**) and Land use land cover classification within the 500 m buffer of each measuring station (**b**). The land cover map of the study area was obtained using an object-based image classification approach applying standard nearest neighbour classifier on a Sentinel-2A image, later verified with Google Earth images. The map was generated using QGIS 3.10.0-A Coruña (https://qgis.org/en/site/).

The study was carried out in the city of Würzburg, one of the major cities in Northern Bavaria with 126,000 inhabitants and one of the driest and warmest city in Southern Germany with a substantial UHI effect^37^. The climate of Würzburg is classified as Cfb by Köppen and Geiger^38^. The annual mean temperature for the period 1965–2015 is 9.6 °C with a temperature range from 1 °C (January) to 20 °C (July) and an annual precipitation amount of 599 mm. Winter is comparatively dry with 43 mm in January whereas the maximum rainfall with 65 mm occurs in July^39^. Seven complete meteorological stations along a transect of six km starting from the city centre (Marktplatz) to sub-urban areas (Gerbrunn) were installed to represent the spatial distribution of built and natural environments (Fig. 1). The sites were selected to represent different land cover types (centre of the city with or without trees, grass lawns, open spaces, paved surfaces and building density) towards the sub-urban area.

### Land use classification

For the classification process and analysis of different LULC classes, we used Sentinel-2A satellite image taken on May 16, 2020 with a cloud cover of only 0.089% (acquired and processed by European Space Agency, ESA). The processing level of the downloaded image was Level-1 C, which includes radiometric and geometric correction, ortho-rectification and spatial registration on a global reference system, namely, the WGS84 datum and Universal Transverse Mercator (UTM) Projection. An image with 10 m resolution (blue, green, red and near infrared) was considered and clipped within the study area as the downloaded image was of 100 km × 100 km size. Aerial imagery and data from Google Earth were used for verification and finally the land use land cover map was determined within a 500 m buffer around the selected seven sites (cf. Fig. 1a).

The digital satellite images were processed using the Trimble eCognition 10 software package. Classification was carried out using object based image analysis (OBIA). In this study, the land use was classified into greenspaces, bare soil, built up open surfaces, roads, buildings and water bodies. For classification of the study area, the standard nearest neighbour classifier was used. Rule sets based on spectral bands were developed in the classifier to classify the study area into the defined classes^40^. Accuracy assessment was also performed (overall accuracy was 96%) and the Kappa coefficient was calculated as 0.9 (total no. of samples = 463). Finally, the area under each class within a 500 m buffer was calculated and then represented in histograms as shown in Fig. 1b.

### Meteorological data collection

The study was carried out between January 1, 2018 and December 31, 2020 by continuously measuring meteorological variables along the urban-sub-urban transect. The year 2018 was one of the warmest years with intense and long lasting heat waves globally^41^ as well as in Germany^39^ followed by 2019 with a comparatively warmer summer and a milder winter. On the other hand, 2020 in comparison was a year that was more close to the long-term average climatic values^39^.

Moreover, to facilitate the understanding of the seasonally varying effects, we analysed the data of summer and autumn, winter and spring together. The categorization of seasons were based on the reports of progressive European summer warming which might favour heat waves to occur in early autumn by 2071–2100^42^. In contrast, the investigation of climate trends in different cities of Bavaria in Southern Germany indicate a rise in mean summer air temperature, but not during winter^43^.

**Figure 2:**
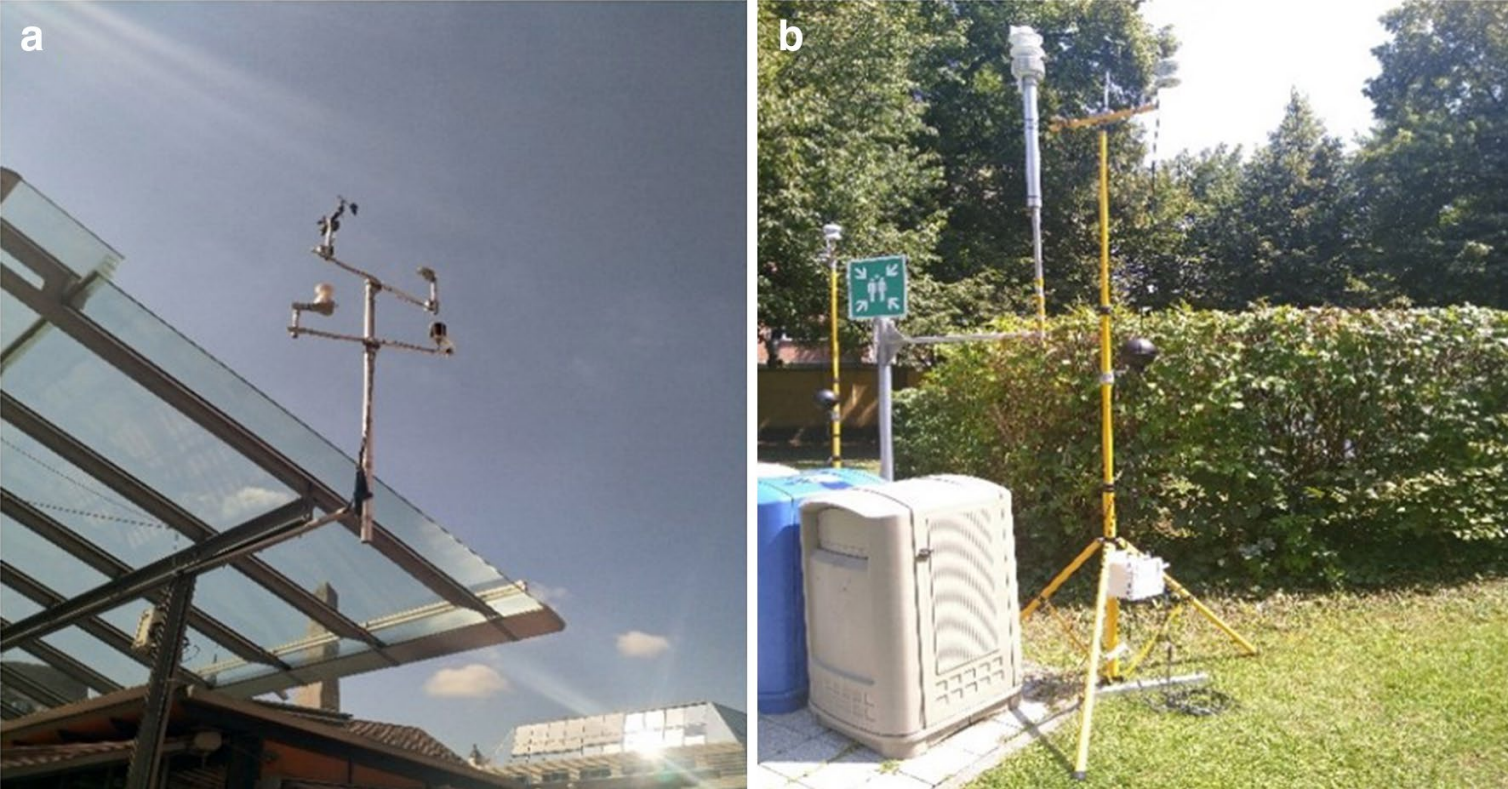
Meteorological station at the centre site—Marktplatz (**a**) and the mobile weather station with black globe temperature logger used to validate the WBGT calculations using the meteorological data (**b**).

At each site, we measured air temperature, relative air humidity and precipitation by installing Campbell CS215 logger and Campbell ARG100 rain gauge stations at a height of three meter from the ground. At the same time, air pressure, wind speed and direction, global radiation were measured by installing Vaisala—Barocap PTB110, RM Young—Wind sentry model 03002-5 and Campbell CS300 Pyranometer at a height of 10 m from the ground (Fig. 2a). The stations were carefully installed at least 10 m apart from trees and buildings to avoid shadowing. All the data were recorded continuously in a 10-min resolution from January 1, 2018 to December 31, 2020 on Campbell CR300 data logger and was collected remotely using a FTP-client.

The (outdoor) WBGT was calculated using the sum of the natural wet bulb temperature *Tw,* the globe temperature *Tg*, and the dry bulb (ambient) temperature *Ta* (Eq. 1) ^44^:

1 $$WBGT=0.7Tw+0.2 Tg+0.1 Ta$$

To calculate WBGT the R programme software packages wbgt.Liljegren of the HeatStress were used. The meteorological data air temperature, relative humidity, solar radiation, wind speed and air pressure as well as information about the exact location of the measuring site were used to calculate *Tg* (Eq. 2), and T_w_ as a function of the mentioned variables following Liljegren et al. ^44^

2 $$T_{g}^{4} = \frac{1}{2}\left( {1 + \varepsilon_{a} } \right)T_{a}^{4} - \frac{h}{{\varepsilon_{g} \sigma }}\left( {T_{g} - T_{a} } \right) + \frac{S}{{2\varepsilon_{g} \sigma }}\left( {1 - \alpha_{g} } \right)\left[ {1 + \left( {\frac{1}{2\cos (\theta )} - 1} \right)f_{dir} + \alpha_{sfc} } \right]$$

here, ε_g_ = 0.95, α_g_ = 0.05, and α_sfc_ = 0.45, h is the convective heat transfer coefficient, S is the solar constant, θ is the solar zenith angle, ${f}_{dir}$ (Eq. 3)

3 $${\text{f}}_{dir} = \left\{ {\begin{array}{*{20}l} {\exp \left( {3{ } - 1.34S^{*} - \frac{1.65}{{S^{*} }}} \right)\theta { }} \hfill & { \le 89.5^\circ } \hfill \\ {0,} \hfill & { > 89.5^\circ } \hfill \\ \end{array} } \right.$$

S^∗^  = S/S_max_; S_max_ is the maximum solar irradiance that would be received in the absence of the atmosphere, S_max_ = S_0_ cos (θ)/d^2^, θ ≤ 89.5°; S_0_ is the solar constant (= 1367 W m^−2^); θ is the solar zenith angle; and d is the Earth–Sun distance in Astronomical Unit.

To validate the calculation of WBGT two sets of mobile weather stations (onset, MA, USA) with globe thermometers were mounted over two tripods. They were set close to a meteorological station (of the same configuration as in the experimental sites in Würzburg) at a centre site (Nordbad) in the city of Munich (Germany) during two warm and dry days (July 7–9, 2020) (Fig. 2c). Firstly, the meteorological variables measured from the mobile weather stations were checked for accuracy using the meteorological data obtained from the meteorological station. Afterwards, the globe temperature (Tg) was validated using the measured and modelled data (Fig. S1).

The mobile weather stations have the specifications shown in Table S1. All sensors of one tripod were connected to a 15 Channel HOBO U30 USB Weather Station Data Logger to log every 10-min average data of 5 s of recordings. The modeled data showed a good agreement with the measured data (Fig. S1).

### Statistical analyses

The collected data were aggregated into hourly mean values (as well as maximum and minimum), daily, seasonal (warm—summer (June–August) and autumn (September–November), cold—winter (December–February) and spring (March–May)) and annual averages were calculated. The 3 years were divided into dry warm years (2018, 2019) and a normal year (2020). Based on the aggregations, we analysed the pattern of WBGT at different stations for the whole year and highlighted the differences of the possible human discomfort at the monitoring sites during conditions of heat stress (maximum air temperature > 30 °C) as well as cold spells (minimum air temperature < 0 °C). Furthermore, we investigated the differences between weather conditions during night and day, to uncover a possible lack of cooling during the night in the city and its effect on WBGT. In this study, air temperature and humidity, wind speed, solar radiation and WBGT were compared applying correlation and linear regression analyses, as well as with two sample *t*-test (Student’s *t*-test). Differences in means were considered significant when p < 0.05. The meteorological variables (n = 157,681 for each variable) as well as the WBGT calculated were subjected to the usual visual residual diagnostics. There was no violation of variance homogeneity. Likewise, normality of errors was verified by making normal q–q plots of the residuals.

To highlight our findings and simplify the understanding of our graphs, we defined Marktplatz as the reference site to which we built the deltas (e.g. $\Delta WBGT = WBGT\;station\;x - WBGT\;Marktplatz$) in order to show the deviation from the city center in degree Celsius. This would result, e.g., in negative delta values for WBGT values lower than the WBGT of the reference site. For statistical analyses and calculations, software R and Excel were used complementarily.

Sensitivity analysis allowed the identification of the parameters that are important and have the greatest influence on the model output, thereby providing useful insight into which model input parameter contributes to the variability of the model output. The method for conducting sensitivity analysis is broadly divided into local and global approaches^45^. Local sensitivity analysis is focused to obtain the sensitivity of a single parameter at a time. Therefore, interaction of different parameters is not taken into consideration. Here we used Sobol sensitivity method, to do an in-depth global sensitivity analysis, since this method is able to quantify not only the contributions of individual parameters, but also their interactions, which could not be obtained as accurately from ANOVA^46^. It is based on the decomposition of model output variance into relative contributions from individual parameters and parameter interactions, the result of which gave us the sensitivity of a given parameter quantified by the ratio (ranging from 0 to 1) of its contribution to the output variance.

The Sobol sensitivity analysis was conducted using R 3.5.0 (R Core Team, 2018), sensitivity (v1.25.0) package, which provides a set of functions useful for performing sensitivity analysis. Daily maximum values over the time span of the study period (further broken down into summer and winter periods of each year) of the meteorological variables (air temperature, relative humidity, wind speed) and the land cover information of greenspace, impervious surfaces and buildings were taken as model inputs against max WBGT as model output.

## Results

### Meteorological differences across the transect

Since all the meteorological stations were installed in such a way that they are not affected by shade at any time of the day, the global radiation was not different between different sites. On average, relative humidity (RH) and wind speed (WS) of the suburban sites (Rottendorfer Straße, Gerbrunn and LGS) were substantially higher and air temperature (AT) was considerably lower compared to the urban sites (Marktplatz, Zu-Rhein-Straße, Paradeplatz and Rennweg) over the 3 years of investigation. Within the urban sites, Marktplatz showed considerably higher AT and WS (the sequence was Marktplatz > Paradeplatz > Rennweg > Zu-Rhein-Straße) and lower RH (the sequence was Rennweg > Zu-Rhein-Straße > Paradeplatz > Marktplatz) (Table 1 and Fig. S2). Within the suburban sites, Gerbrunn and LGS showed lower AT but higher RH and WS compared to Rottendorfer Straße.

**Table 1:** Minimum (min), average ($\overline{x }$) and maximum (max) values of air temperature (AT), relative humidity (RH), wind speed (WS) and global radiation of urban and sub-urban sites over 3 investigated years (± SE of mean values between years).

Variables	Seasons	Stats	Urban sites				Sub-urban sites		
			Marktplatz	Paradeplatz	Rennweg	Zu-Rhein-Straße	Rottendorfer Straße	LGS	Gerbrunn
AT	Warm	Min	− 2.9 ± 1.3	− 2.4 ± 1.2	− 3.3 ± 1.2	− 3.5 ± 1.2	− 3.4 ± 1.3	− 4.1 ± 1.4	− 4.8 ± 1.4
		$\overline{x }$	16.7 ± 0.3	16.6 ± 0.3	16.3 ± 0.3	16.2 ± 0.3	15.7 ± 0.3	15.4 ± 0.3	15.6 ± 0.2
		Max	37.9 ± 1.1	37.3 ± 0.9	37.7 ± 1.0	36.8 ± 0.8	36.2 ± 0.8	37.3 ± 0.8	36.5 ± 0.9
	Cold	Min	− 5.5 ± 1.4	− 7.6 ± 1.7	− 8.1 ± 1.8	− 8.3 ± 1.9	− 8.6 ± 1.8	− 9.2 ± 1.8	− 6.8 ± 1.7
		$\overline{x }$	9.3 ± 1.5	7.9 ± 0.3	7.6 ± 0.3	7.5 ± 0.3	7.0 ± 0.3	6.7 ± 0.3	7.1 ± 0.4
		Max	28.6 ± 1.3	28.2 ± 1.5	28.4 ± 1.6	27.5 ± 1.5	27.2 ± 1.6	27.4 ± 1.7	27.5 ± 1.5
RH	Warm	Min	18.1 ± 1.3	19.0 ± 1.4	18.0 ± 1.2	19.5 ± 1.5	20.0 ± 1.8	14.8 ± 5.0	7.1 ± 4.6
		$\overline{x }$	67.6 ± 1.8	68.5 ± 1.8	70.8 ± 1.9	70.6 ± 1.8	71.2 ± 1.8	72.2 ± 1.8	72.5 ± 1.9
		Max	99.9 ± 0.0	99.9 ± 0.1	100.0 ± 0.0	100.0 ± 0.0	100.0 ± 0.0	100.0 ± 0.0	100.0 ± 0.0
	Cold	Min	16.8 ± 0.6	16.8 ± 0.6	17.4 ± 0.9	17.9 ± 0.6	18.7 ± 0.8	18.5 ± 0.9	15.5 ± 8.5
		$\overline{x }$	71.0 ± 1.0	72.6 ± 0.6	75.5 ± 0.6	75.4 ± 0.8	76.4 ± 0.8	76.6 ± 0.7	79.9 ± 2.5
		Max	99.9 ± 0.1	100.0 ± 0.0	100.0 ± 0.0	100.0 ± 0.0	100.0 ± 0.0	100.0 ± 0.0	100.0 ± 0.0
WS	Warm	Min	0.0 ± 0.0	0.0 ± 0.0	0.0 ± 0.0	0.0 ± 0.0	0.0 ± 0.0	0.0 ± 0	0.0 ± 0
		$\overline{x }$	0.8 ± 0.1	0.7 ± 0.0	0.5 ± 0.0	0.3 ± 0.0	0.7 ± 0.0	0.9 ± 0.0	1.3 ± 0.0
		Max	5.3 ± 0.3	7.7 ± 0.4	5.3 ± 0.6	4.0 ± 0.4	4.8 ± 0.1	6.3 ± 0.5	7.6 ± 0.4
	Cold	Min	0.0 ± 0.0	0.0 ± 0.0	0.0 ± 0.0	0.0 ± 0.0	0.0 ± 0.0	0.0 ± 0	0.0 ± 0
		$\overline{x }$	1.0 ± 0.0	0.9 ± 0.0	0.8 ± 0.0	0.7 ± 0.0	1.3 ± 0.0	1.5 ± 0.0	1.7 ± 0.0
		Max	5.7 ± 0.4	8.6 ± 0.5	6.6 ± 0.5	5.0 ± 0.0	6.6 ± 0.4	8.9 ± 0.5	9.9 ± 1.2
Radiation	Warm	Min	0.0 ± 0.0	0.0 ± 0.0	0.0 ± 0.0	0.0 ± 0.0	0.0 ± 0.0	0.0 ± 0.0	0.0 ± 0
		$\overline{x }$	139.0 ± 6.1	122.5 ± 4.9	141.7 ± 4.8	111.8 ± 3.1	146.4 ± 4.9	152.5 ± 6.0	160.2 ± 4.2
		Max	987.0 ± 36.0	1010 ± 38.3	1000.7 ± 18.6	979.7 ± 22.2	1004.7 ± 20.0	1070.0 ± 19.1	1071.3 ± 13.9
	Cold	Min	0.0 ± 0.0	0.0 ± 0.0	0.0 ± 0.0	0.0 ± 0.0	0.0 ± 0.0	0.0 ± 0.0	0.0 ± 0
		$\overline{x }$	109.4 ± 15.1	83.6 ± 3.5	100.4 ± 3.5	82.7 ± 82.7	102.9 ± 3.7	103.2 ± 3.7	99.0 ± 10.2
		Max	978.7 ± 17.5	978.0 ± 2.6	978.0 ± 4.9	944.7 ± 21.1	996.0 ± 35.7	1015.0 ± 26.1	1067.0 ± 26.5

However, during the normal year 2020, the difference between the urban sites was considerable in terms of RH and WS, but not in terms of AT. Whereas, during the dry years, in particular during 2018, AT was significantly higher at Marktplatz (mean, $\overline{x }=15.5$°C) compared to Paradeplatz ($\overline{x }=12.6$°C, p = 4.70E − 06), Rennweg ($\overline{x }=12.4$°C, p = 7.09E − 07) and Zu-Rhein ($\overline{x }=12.2$°C, p = 1.95E − 07). At the same time, RH was significantly lower at Marktplatz ($\overline{x }=66$%) compared to Zu-Rhein ($\overline{x }=72$%, p = 3.99E − 07), Rennweg ($\overline{x }=71$%, p = 8.45E − 07) and Paradeplatz ($\overline{x }=69$%, p = 0.00470).

### WBGT over spatial and temporal scales

**Figure 3:**
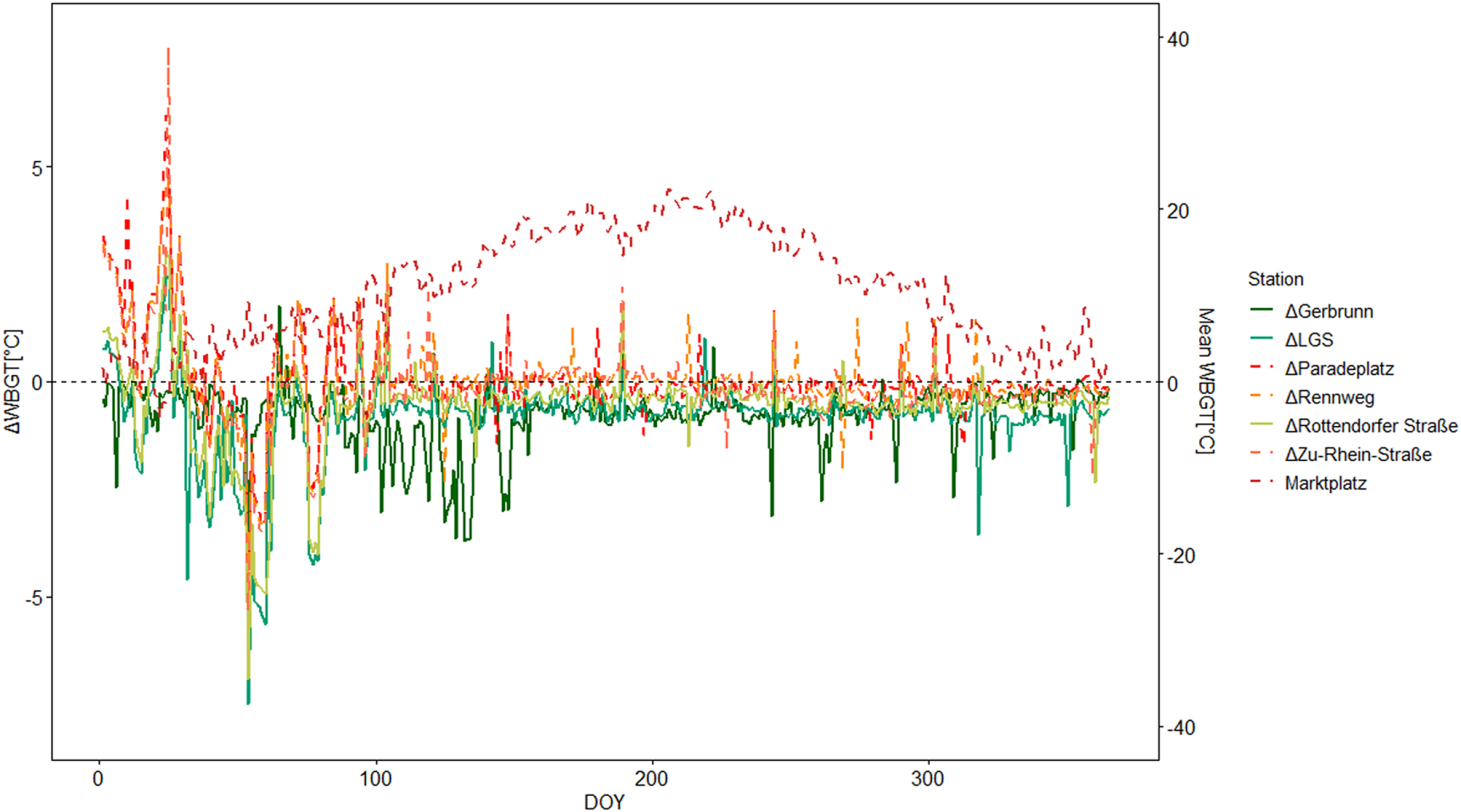
Three years average WBGT of Marktplatz and the average difference of WBGT in relation to Marktplatz (0-line as reference) at seven sites.

On average, over the 3 years of investigation, the centre location Marktplatz showed the highest WBGT values while suburban sites had substantially lower values (Fig. 3). However, the other urban sites did not vary considerably compared to Marktplatz. Separating the years into dry and warm (2018 and 2019) and normal years (2020), the urban sites indicated significantly lower values during the dry years compared to the reference site Marktplatz (Paradeplatz, p = 0.00058, Rennweg, p = 0.00096 and Zu-Rhein-Straße, p = 0.00014).

**Figure 4:**
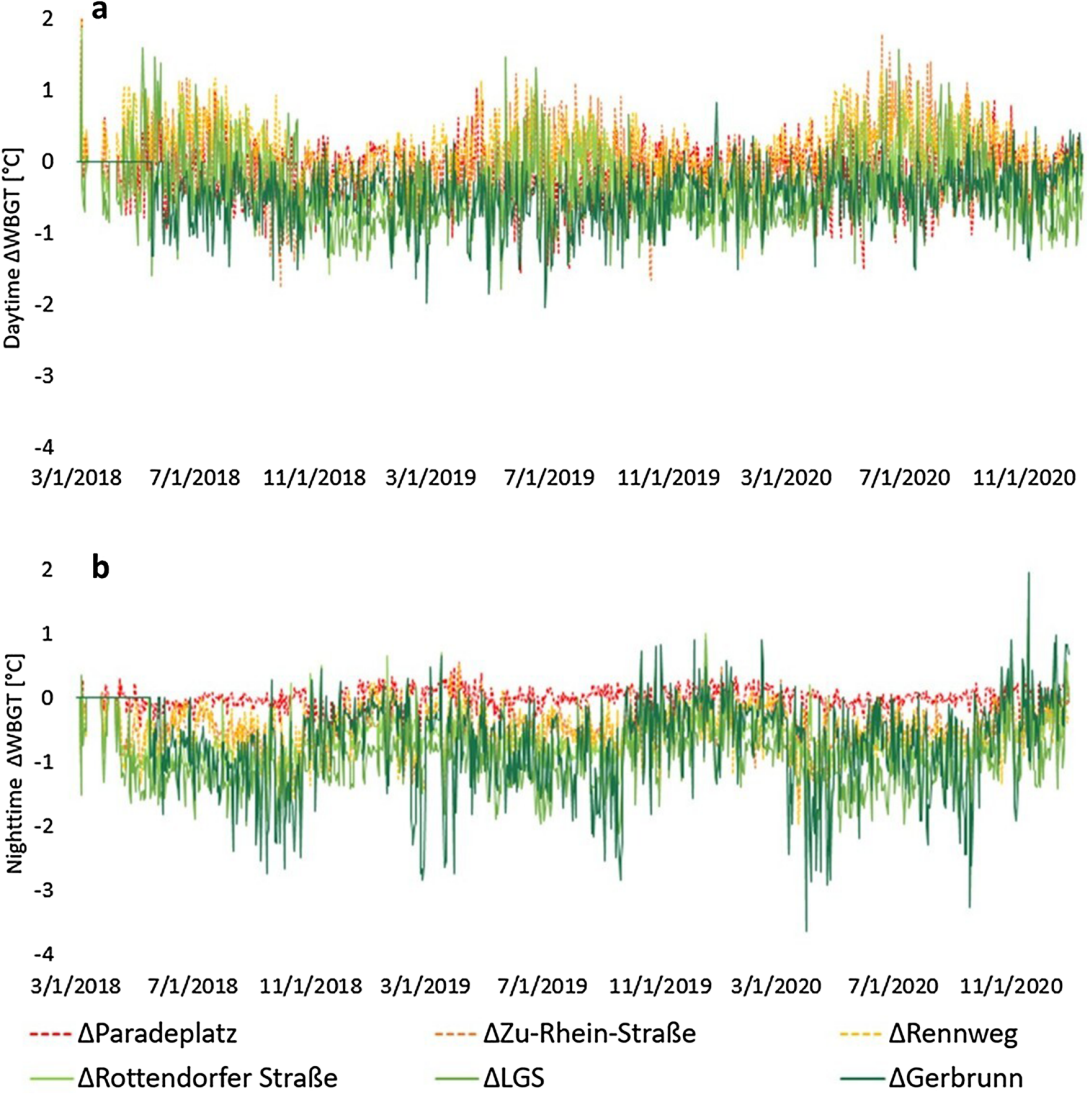
Differences (∆) of average WBGT of urban and sub-urban sites during day (**a**) and nighttime (**b**) compared to the centre site Marktplatz over 3 years (2018–2020).

Considering the diurnal pattern, both during day and night time, sub-urban sites were substantially cooler than urban sites (Fig. 4). During the daytime, Gerbrunn and LGS were around 0.5 °C and Rottendorfer Straße around 0.3 °C cooler compared to Marktplatz. During the nighttime, LGS were around 1 °C, Gerbrunn around 0.8 °C and Rottendorfer Straße around 0.7 °C cooler than the central sites Marktplatz and Paradeplatz. Among the urban sites at night, Rennweg and Zu-Rhein-Straße were around 0.5 °C cooler than Marktplatz and Paradeplaltz (Marktplatz and Paradeplatz were equally warm).

**Figure 5:**
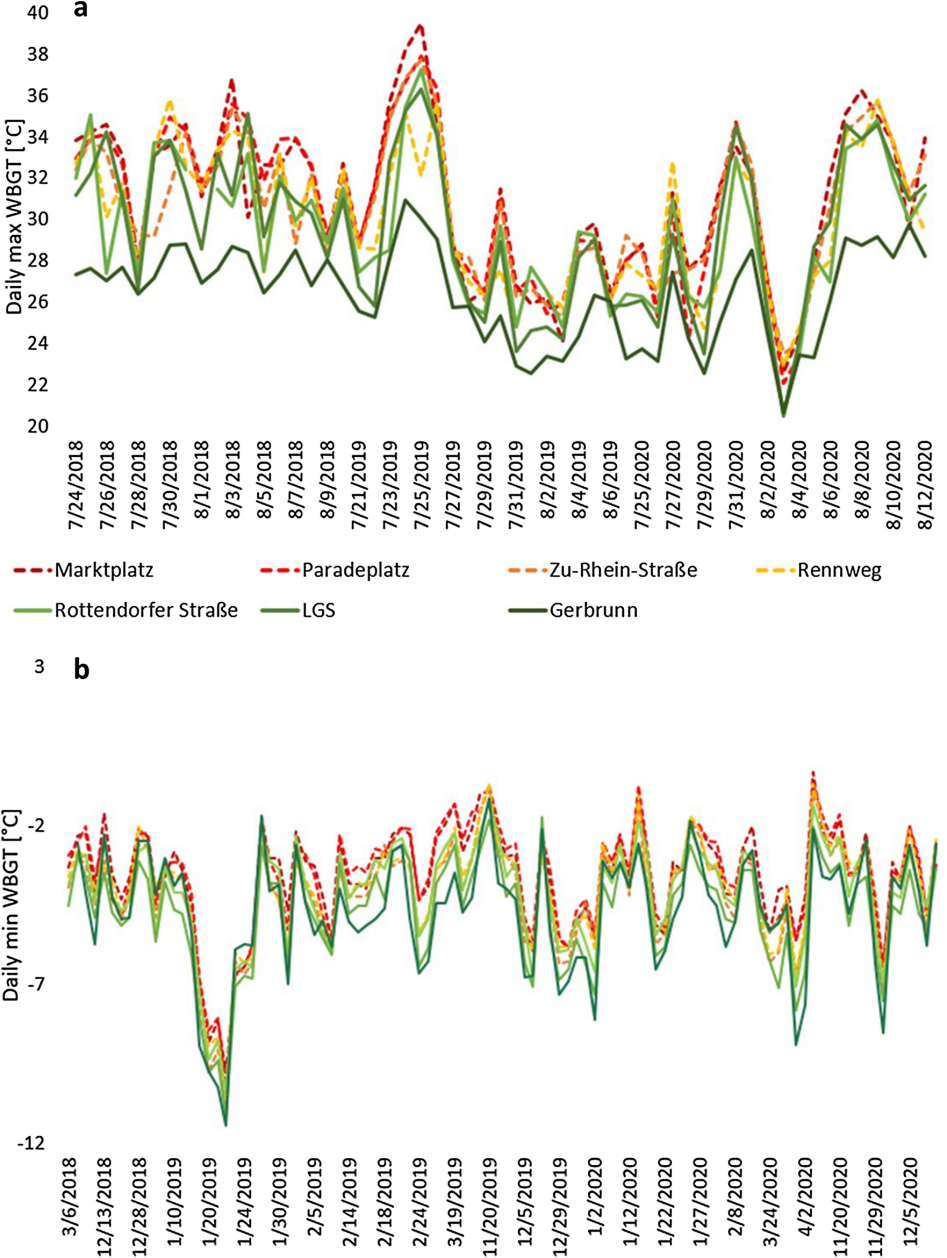
Daily maximum WBGT at seven sites in Würzburg during the hottest weeks over 3 investigated years (2018–2020) (**a**) and minimum WBGT during the cold days (minimum air temperature < 0 °C) (**b**).

While analyzing the daily maximum WBGT during hot days (maximum air temperature > 30 °C) over 3 years, suburban sites were on average significantly cooler with WBGT values of 30.4 °C at the LGS, p = 0.00000, 30 °C at the Rottendorfer Straße, p = 0.00059 and 27 °C in Gerbrunn, p = 0.00024 compared to 31.6 °C at Marktplatz (Fig. 5). Within the urban sites only Rennweg showed significantly lower WBGT with 30.7 °C, p = 0.01183. The difference between Paradeplatz (31.5 °C) and Zu-Rhein-Straße (31 °C) was not significant.

During cold days (minimum air temperature < 0 °C), no significant differences were found between the urban sites in terms of average minimum WBGT (− 3.6, − 3.6, − 4.0 and − 4.1 °C for Marktplatz, Paradeplatz, Rennweg and Zu-Rhein-Straße respectively). However, suburban sites were significantly cooler compared to the reference site Marktplatz with minimum WBGT (− 4.7, − 4.7 and − 4.1 for Gerbrunn, p = 2.53E − 05, LGS, p = 6.01E − 06, and Rottendorfer Straße, p = 0.02729 respectively) (Fig. 5).

### Relationship between land covers and WBGT during the hot and cold days

**Figure 6:**
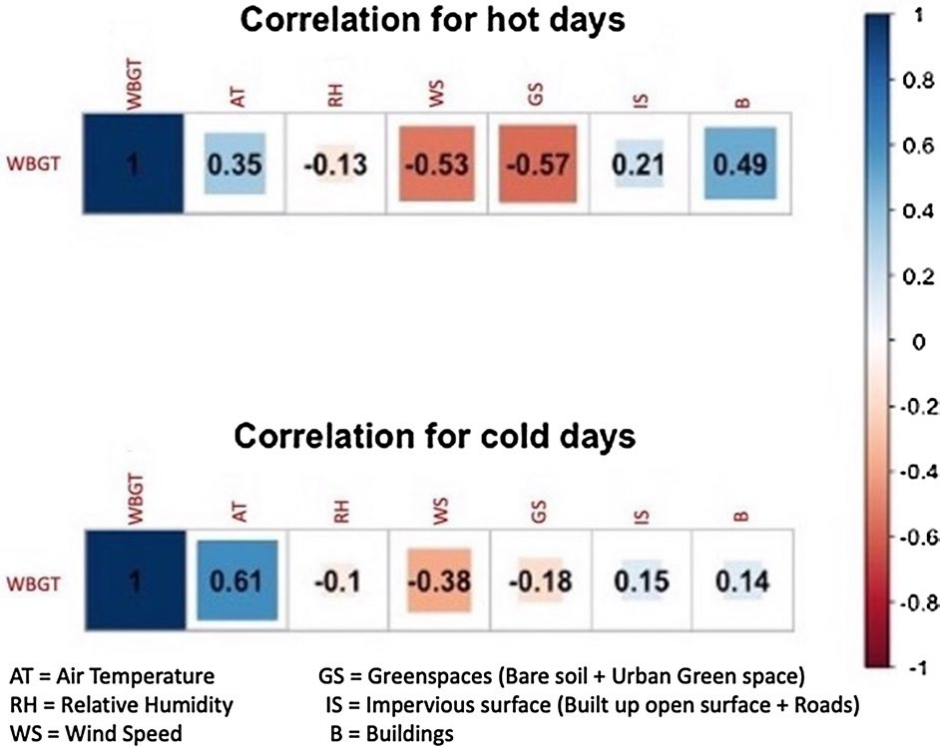
Pearson correlation coefficient (r) between meteorological variables and land cover types with WBGT.

The analysis of the relationship between meteorological variables, land cover types and outdoor thermal stress (WBGT) during hot and cold days showed that during hot summer days wind speed (WS) had the highest negative effect followed by relative humidity (RH), whereas air temperature (AT) showed a positive correlation with WBGT (Fig. 6). In terms of land cover components (Fig. 1b), percentage of the combined cover of urban greenspace, bare soil and water body within the total 500 m buffer area showed a strong negative correlation (r = − 0.57) compared to a moderate to strong positive relations of buildings (B) and impervious surfaces (IS, including built up open surfaces, roads) (r = 0.49 and r = 0.21 respectively) (Fig. 6).

During the cold winter days, the correlation coefficient of most of the meteorological variables and land cover types were comparatively weak except for the air temperature (Fig. 6). WS and RH still showed negative impact and AT showed almost double positive effect (r = 0.61) in influencing the WBGT. Within the land cover types, B and IS showed a weak positive association in terms of increasing WBGT (r = 0.14 and r = 0.15) compared to a moderate negative impact of GS (r = − 0.18) (Fig. 6).

### Sensitivity of different land cover types over WBGT

Sensitivity analysis showed a clear influence of greenspaces, buildings and impervious surfaces within the 500 m buffer of the meteorological stations on the reduction of WBGT during summer and winter (Fig. S3). However, the influence was sharply contrasting depending on the climate conditions. During the warm and dry summer of 2018, GS was almost four times more influential compared to the summer days of 2019 and 2020. Buildings and impervious surfaces constantly showed considerable influence on WBGT during all of the investigated years.

During the winter, in contrary, both GS and B showed substantial influence on WBGT during comparatively colder winter of 2019 in relation to mild winter of 2018 and 2020. Within the built environment, buildings showed a more prominent influence on WBGT compared to impervious surfaces.

Unexpectedly, the impact of buildings was not so clear during the winter of 2020 and of IS during the summer of 2019.

## Discussion

Experimental data collected over three consecutive years including dry and warm summers and winters clearly showed that the magnitude of UHI intensity follows the spatial pattern of land cover types. The centre of the city with less greenspaces and more built surfaces was warmer during both summer and winter compared to the sub-urban areas. The discrepancies were larger during warmer summers and cold winter days. Even though buildings also provided shade, the deflection of wind flow reduced human thermal comfort compared to greenspaces. Greenspaces with their higher albedo and shading effect along with the boundary layer cooling through transpiration have been widely investigated ^47–49^ in previous studies as a mitigation measure in terms of size^4,50^, type^51,52^, shape, location and layout^13,33^. However, effectiveness of those mitigation measures for outdoor heat and cold stress over seasons in terms of land cover types are mostly overlooked^20,53^.

The amount of greenspaces and bare soil (GS) decreases continuously from the sub-urban (Gerbrunn) to the central urban (Marktplatz) sites. Correspondingly, the proportions of buildings increased from the sub-urban to urban sites (Fig. 1b). A clear relationship was shown to exist between a higher percentage of GS with lower air temperature and the increase of relative air humidity. Wind speed also showed a reasonable correlation with land cover, especially considering the amount of GS that increased wind speed whereas buildings can actually reduce the windspeed. In particular, these differences were much stronger during the hot and dry years as seen within the urban sites during the year 2018. On average, during the warm summers, air temperature of city centres were 1.3 °C and during cold winters, up to 5 °C warmer than the sub-urban areas.

Urban sites showed higher wet-bulb globe temperature (WBGT) compared to the sub-urban sites both during summer and winter time. Similarly, previous studies such as Molenaar et al. ^54^ showed that heat stress was almost double in a city compared to that in the countryside. During the hot days, a difference of close to 8 °C WBGT was observed between Marktplatz (37.8 °C) and Gerbrunn (31.7 °C). This is particularly dangerous for humans since WBGT above 35 °C might not allow air circulation, which could remove heat from the body^41^. Out of 97 hot days (maximum air temperature > 30 °C) over 3 years of investigations, at the centre urban site Marktplatz and Paradeplatz there were 9 days that reached this extreme threshold of WBGT > 35 °C compared to 6 days each at Rennweg and Zu-Rhein straße. On the other hand, sub-urban sites Rottendorfer straße had four and LGS had 3 days with no extreme hot day at Gerbrunn. It is interesting to note that, even having the similar amount of greenspaces in Zu-Rhein-Straße and Rennweg within the 500 m buffer, it was mainly the building proportion which cause the reduced wind speed and lower WBGT values. Presence of compact urban structures with high-rise buildings affect the aerodynamics and microscale wind conditions^34^. Hence, the cooling range of greenspaces is not only dependent on the size and shape of greenspaces^55^ but also on the morphology of the built up area surrounding the greenspaces^56^. Therefore, during the summer days, the building heights and the proportion of land cover can significantly increase the WBGT.

On the other hand, during cold days, sub-urban sites were significantly colder compared to the urban sites. There were no significant differences regarding WBGT within the urban sites. Nevertheless, the succession of more built-up areas and buildings and less green areas showed effects on the extent of outdoor thermal stress. Trees can act as a wind barrier, thus a heat sink druing cold days^55^; however, grass lawns and bare soils could potentially allow higher radiation access^36^. The GS (including open grass lawns) in our study indicate the combined effect—relatively weaker correlations of GS with WBGT during cold days. Similarly, the sites LGS and Gerbrunn with wide open grass lawns showed better night-time cooling compared to Rottendorfer Straße with tall trees. More open space is preferred for night-time cooling as well as for winter solar radition.

From correlation analyses of meteorological variables and the landcover types it became clear that windspeed is one of the major driver for outdoor thermal comfort assessment followed by air temperature, both during summer and winter time. Our results also support the importance of these factors as shown by comparatively higher values of WBGT at Zu-Rhein-Straße compared to Rennweg within the urban sites. Similarly, having almost identical amount of built up areas and buildings but with slightly higher greenspace might explain the significantly higher air temperature during the hot year 2018 at Marktplatz compared to Paradeplatz and consequently the slightly higher WBGT. Similarly, greenspaces can reduce the outdoor thermal comfort during cold winter days. However, the extent is much lower compared to the beneficial impact during hot summer days. Out of 85 cold days over 3 years of investigations, urban sites had between 1 and 2 days of extreme cold stress (minimum air temperature < 0 °C) with WBGT < − 10 °C compared to sub-urban sites with up to 5 days at Gerbrunn. Therefore, the extreme thermal stress during summer and winter, and the tradeoffs between winter penalties and summer incentives should be considered carefully. Our analyses showed a small effect of built open surfaces (IS) both during summer and winter times, which might be dealt with caution. Moreover, the negative effects of impermeable surfaces on the natural ecosystem functioning such as storm water management and microbial activity are significant^57^. A careful planning of mixed development of built surfaces with greenspaces could potentially create a win–win situation across the seasons providing ecosystem services.

In view of ongoing urbanization and climate change, we need to understand the extent of the impact of both built environment and greenspaces for outdoor thermal comfort. In other words, both summer time penalties and wintertime UHI intensities should be considered while applying the knowledge of urban climatology to integrate into the planning process^20^. If built surfaces cause the UHI, they could also potentially reduce the cold stress during the winter. We found that the risk of extreme summer heat stress could be halved in the presence of 30–40% greenspaces without a little compromise in winter cold stress. However, more empirical research is required to quantify the extent of these tradeoffs. In particular, with the advent of higher resolution aerial or remote sensing thermal images coupled with ground data might improve our understanding of the cooling potential of urban greenspaces in heterogeneous urban environments. However, identical sites with similar built and natural surfaces at a proportional scale and types like in our present study are difficult to get. The effects of one-land cover type therefore might have masked the magnitude of effects of another as reported here.

## Conclusion

The present study allowed the assessment of different land use types and resultant summer heat and cold-wave hazards in order to support their balancing when adopting mitigation measures. We point out that, there is no panacea for balancing summer and wintertime thermal benefits, rather strategically planned greenspaces within open spaces that have to fulfill several, often competing demands are of utmost importance. Having said that, planning of green infrastructure needs to be rather more holistic and consider the effects of dense versus sparse settlement effects on all other ecological (such as storm water management, biodiversity), social (social coherance) and economic (transportation) pillars of sustainability. Definitely, more studies are needed in this regard. From the present study recommendations can be drawn for adaptive planning at two scales—neighbourhood and site scale.

At the neighborhood scale: I. Cooling benefits from small park islands also depends on the character of the surrounding development since wind was identified as the most important parameter for summer thermal comfort in this study. Therefore, a mix of open lawns and trees within the dense city centers will have better impact in optimizing the co-benefits during diurnal and seasonal thermal loads. II. Mixed development compared to the dense city centre will reduce the tradeoffs of summer heat stress and winter heat demand. Both building height and size (amount of land cover) showed significant impact on increasing outdoor thermal stress during hot summer days. Overall, a surface cover of 30–40% greenspace within the built-environment can maximize the summer cooling without little compromise in the winter.

At site scale: I. Greenspaces might compromise the winter thermal comfort a little since convective heat is the most important parameter for winter thermal comfort but it is simply outweighed by the summer cooling benefit of greenspaces. In particular, during the hot summer days, GI showed four times more influence in reducing the outdoor thermal heat stress compared to moderate summer days. Planting trees in the city centre could be the best adaptation strategy and if not possible green façade or green roof also will help. II. At the windward sides, planting small plants or shrubs in open spaces within the city centre and rather big trees within the sub-urban areas will maximize the summer and winter thermal comfort benefits.

## Supplementary Information

Supplementary Information.
