# Supplementary material for: Spatial and temporal changes of outdoor thermal stress: influence of urban land cover types
Source: Sci Rep. 2022 Jan 13;12:671. doi: 10.1038/s41598-021-04669-8 (PMC8758735; doi:10.1038/s41598-021-04669-8)
Supplement: Supplementary file 1 — Supplementary Information. [file 41598_2021_4669_MOESM1_ESM.docx]

**Title: Spatial and temporal changes of outdoor thermal stress: influence of urban land cover types**

# Mohammad A. Rahman^a^*, Eleonora Franceschi^b^, Nayanesh Pattnaik^a^, Astrid Moser-Reischl^b^ Christian Hartmann^c^, Heiko Paeth^c^, Hans Pretzsch^b^,Thomas Rötzer^b^, Stephan Pauleit^a^

^a^Strategic Landscape Planning and Management, School of Life Sciences, Weihenstephan, Technische Universität München, Emil-Ramann-Str. 6, 85354 Freising, Germany

^b^Forest Growth and Yield Science, School of Life Sciences, Weihenstephan, Technische Universität München, Hans-Carl-von-Carlowitz-Platz 2, 85354 Freising, Germany

^c^ Institute of Geography and Geology, Universität Würzburg, Am Hubland, 97074 Würzburg

*Corresponding author, Mohammad A RAHMAN, Email: ma.rahman@tum.de, Tel: +49 (0)8161 71 4661

Email address of co-authors:

Eleonora FRANCESCHI: [eleonora.franceschi@tum.de](mailto:eleonora.franceschi@tum.de)

Nayanesh PATTNAIK^:^ nayanesh.pattnaik@tum.de

Astrid MOSER-REISCHL: [astrid.reischl@tum.de](mailto:astrid.reischl@tum.de)

Christian HARTMANN: [christian.hartmann@uni-wuerzburg.de](mailto:christian.hartmann@uni-wuerzburg.de)

Heiko PAETH: [heiko.paeth@uni-wuerzburg.de](mailto:heiko.paeth@uni-wuerzburg.de)

Hans PRETZSCH: [hans.pretzsch@tum.de](mailto:hans.pretzsch@tum.de)

Thomas RÖTZER: [thomas.roetzer@tum.de](mailto:thomas.roetzer@tum.de)

Stephan PAULEIT: pauleit@tum.de

**Supplementary data**

Table S1: Information of the used sensors and their accuracy to measure the meteorological variables for validation of WBGT calculations.

| Parameter | Sensor | Accuracy |
| --- | --- | --- |
| Wind and gust speed | S-WSB-M003 smart sensors | ± 1.1 m s^-1^ |
| Solar radiation | silicon pyranometer S-LIB-M003 | ± 10 W m^-2^ |
| Air temperature and relative humidity | 12-bit temperature/relative humidity smart sensor S-THB-M002 installed within solar radiation shield RS3-B | ± 0.21 °C, RH: ± 2.5% |
| globe temperature | black globe thermometer (diameter 150 mm) | ± 0.2 °C |


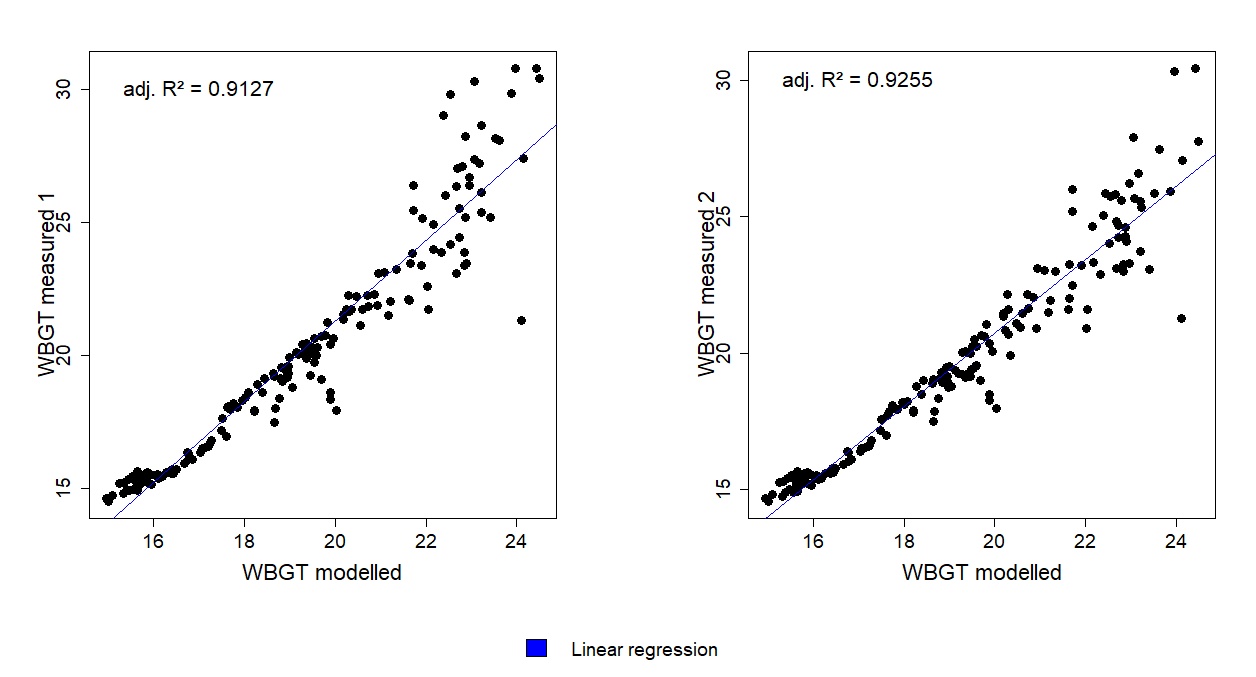


Figure S1: Relationship between measured and modeled values of WBGT


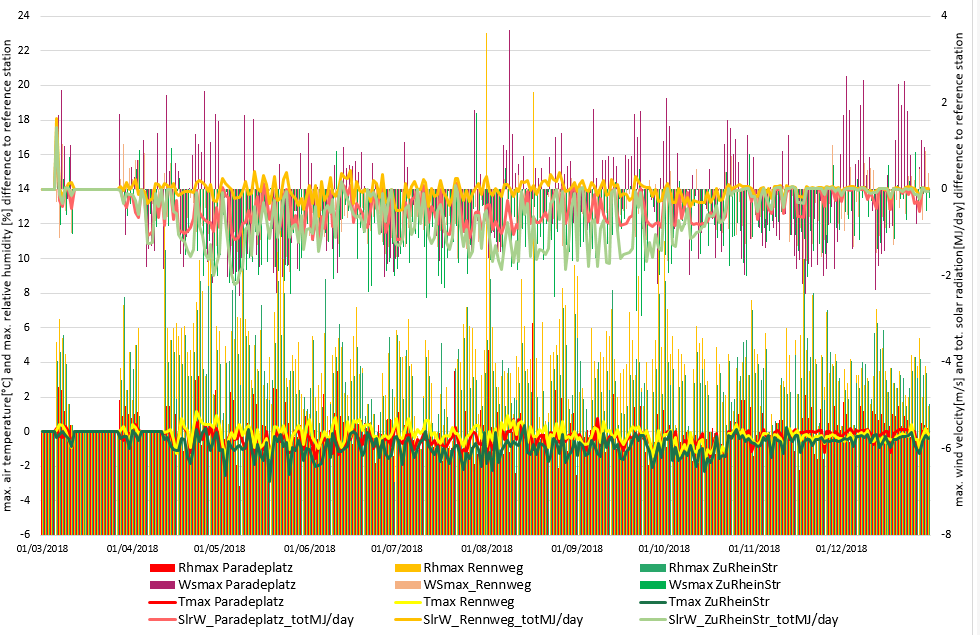


2018 – Dry year

2020 – Normal year


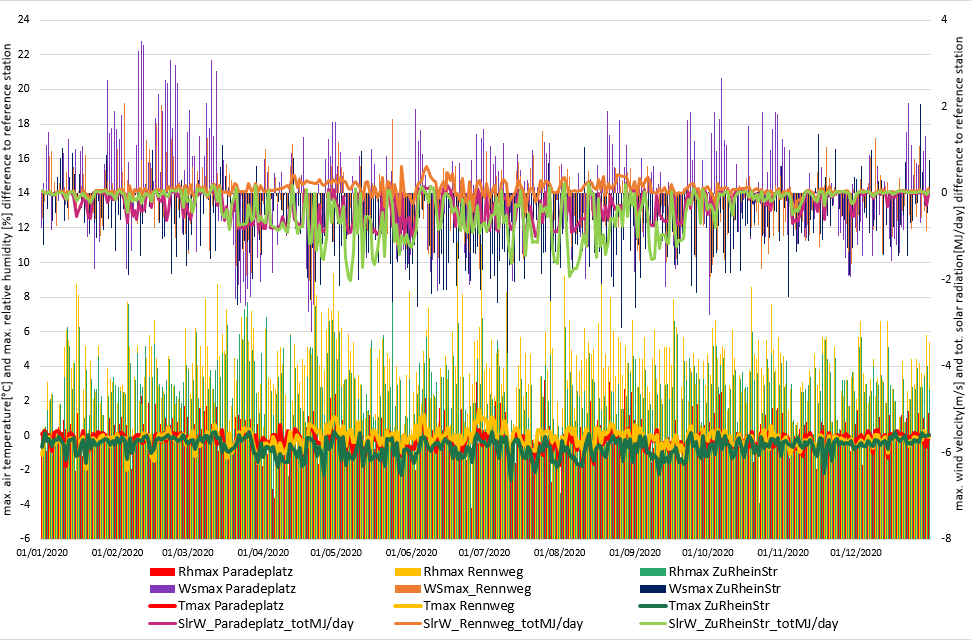

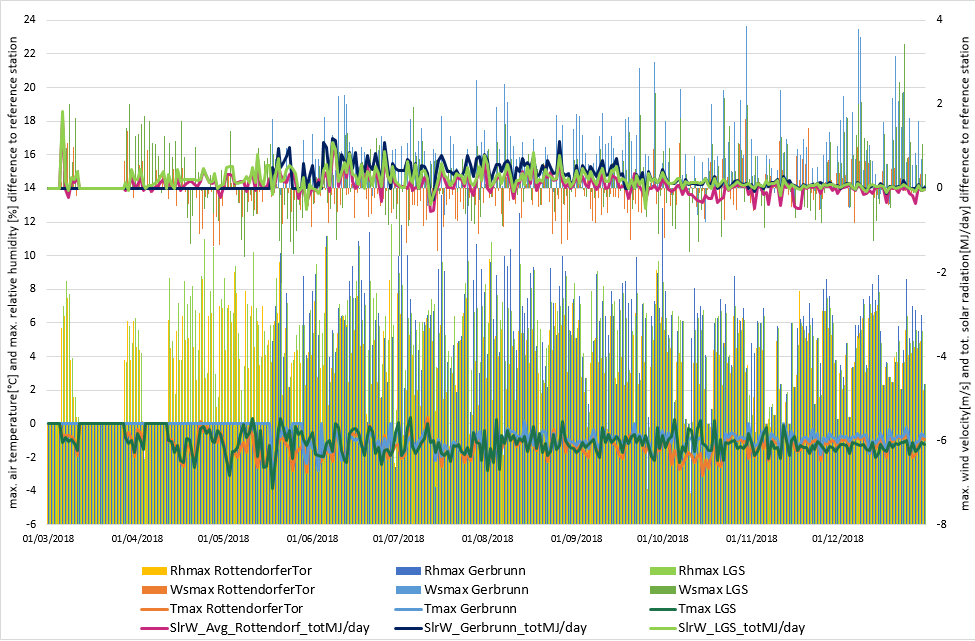

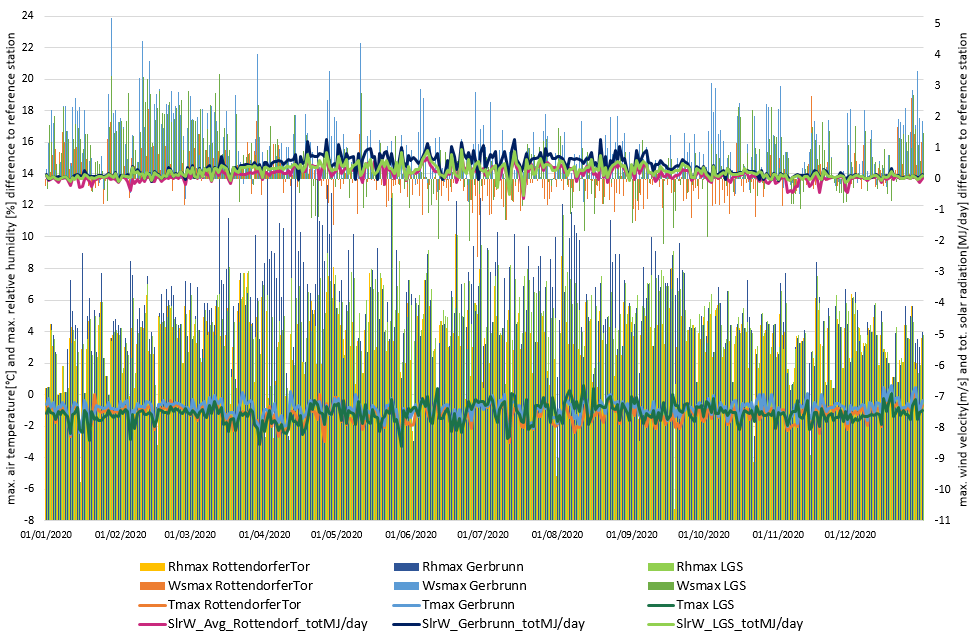


SUBURBAN/RURAL

URBAN

Figure S2: Differences (∆) of maximum air temperature, relative humidity, wind speed and daily total radiation of urban and sub-urban sites compared to the centre site Marktplatz during 2018 and 2020.


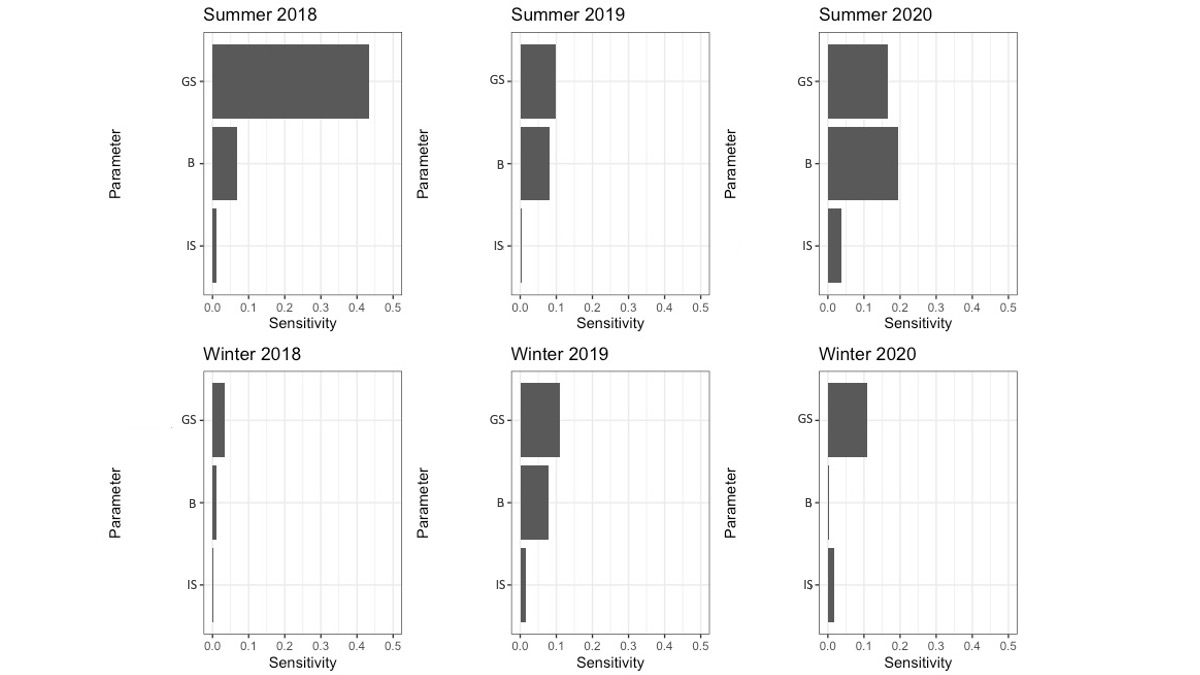


Figure S3: Total effect sensitivity index of three land cover types during summer (summer and autumn) and winter (winter and spring) of 2018, 2019 and 2020.
